# Supplementary figures and images for: Kidney organoids recapitulate human basement membrane assembly in health and disease
Source: eLife. 2022 Jan 25;11:e73486. doi: 10.7554/eLife.73486 (PMC8849328; doi:10.7554/eLife.73486)

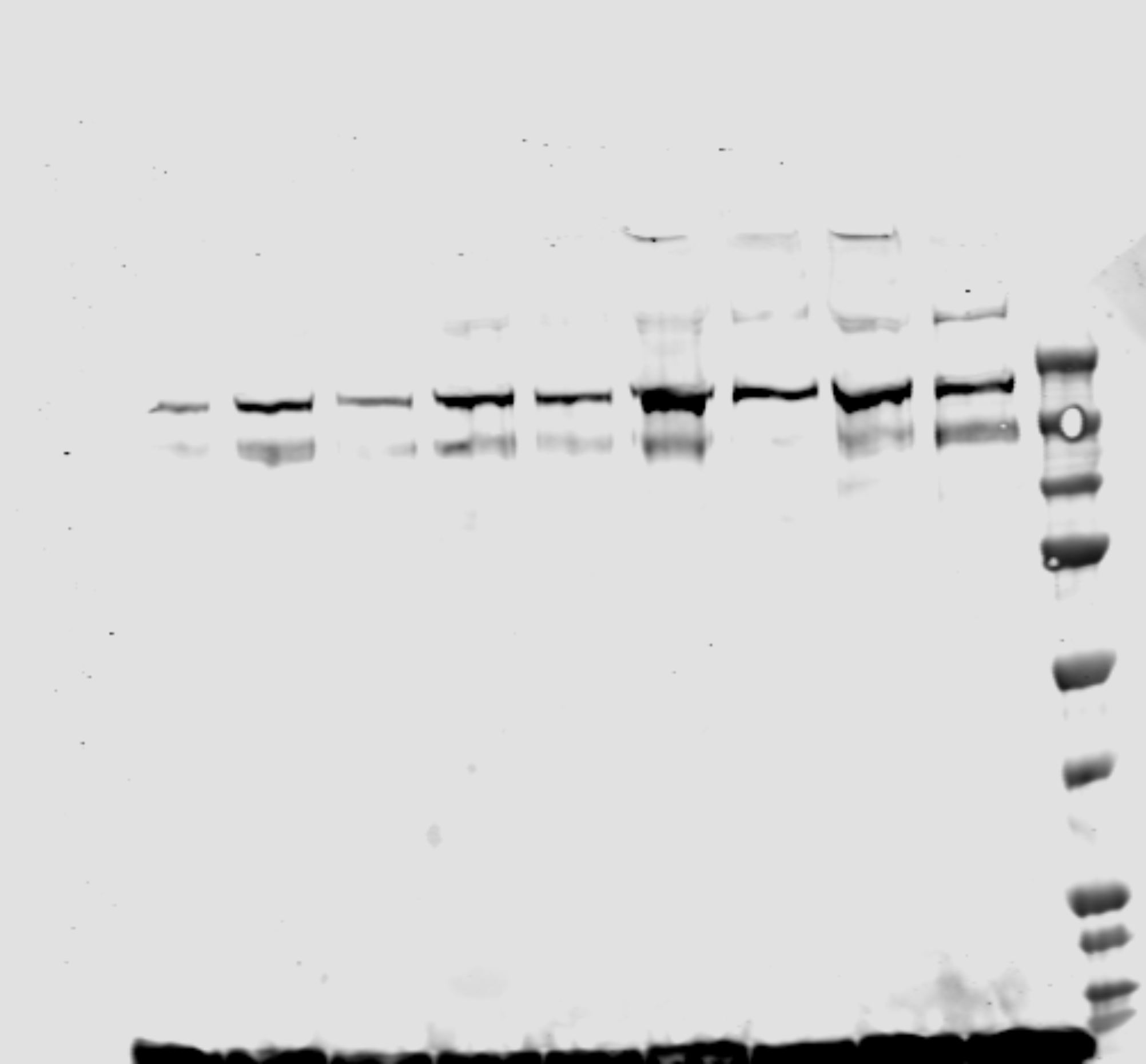

Supplement: Figure 1—source data 1. [file elife-73486-fig1-data1.zip › Fig 1H - source data WB LAMB2.tif]
