# Supplementary material for: Kidney organoids recapitulate human basement membrane assembly in health and disease
Source: eLife. 2022 Jan 25;11:e73486. doi: 10.7554/eLife.73486 (PMC8849328; doi:10.7554/eLife.73486)
Supplement: Transparent reporting form [file elife-73486-transrepform1.docx]

***eLife’s* transparent reporting form**

We encourage authors to provide detailed information *within their submission* to facilitate the interpretation and replication of experiments. Authors can upload supporting documentation to indicate the use of appropriate reporting guidelines for health-related research (see [EQUATOR Network](http://www.equator-network.org/%20)), life science research (see the [BioSharing Information Resource](https://biosharing.org/)), or the [ARRIVE guidelines](http://www.plosbiology.org/article/info:doi/10.1371/journal.pbio.1000412) for reporting work involving animal research. Where applicable, authors should refer to any relevant reporting standards documents in this form.

If you have any questions, please consult our Journal Policies and/or contact us: [editorial@elifesciences.org](mailto:editorial@elifesciences.org).

**Sample-size estimation**

- You should state whether an appropriate sample size was computed when the study was being designed
- You should state the statistical method of sample size computation and any required assumptions
- If no explicit power analysis was used, you should describe how you decided what sample (replicate) size (number) to use

Please outline where this information can be found within the submission (e.g., sections or figure legends), or explain why this information doesn’t apply to your submission:

**Mass spectrometry:** Sample size was determined based on literature guidance and previous studies:

**a) Mouse study** - sample size: n = 6 mouse kidney samples fractionated (Methods, sample enrichment for proteomics analysis, pg. 17), n = 4 mouse kidney samples for laser microdissection microscopy from which 150 (in **Methods**, ***section laser microdissection microscopy*** pg. 17);

**b) Organoid study**: n = 3 differentiation replicates (diff 22, diff 23, diff 24) and 3 time points analyzed (day 14, day 18 and day 25); organoids were pooled as follows (in **Methods**, ***sample enrichment for proteomics analysis***, pg. 17):

|  | Day 14 | Day 18 | Day 25 |
| --- | --- | --- | --- |
| Diff 22 | 17 organoids | 15 organoids | 12 organoids |
| Diff 23 | 20 organoids | 12 organoids | 12 organoids |
| Diff 24 | 18 organoids | 18 organoids | 18 organoids |

**Histology and immunofluorescence:**

**a) Human and mouse study**: for each time-point, we used we used formalin-fixed paraffin-embedded sections from n = 1 human or mouse fetal kidney (in **Methods**, ***Histology and immunofluorescence***, pg. 15);

**b) Organoid study**: for each time-point, we used formalin-fixed paraffin-embedded sections from n = 1 organoid generated from the same differentiation replicate (in **Methods**, ***Histology and immunofluorescence***, pg. 15).

**SDS-PAGE and immunoblotting**: n = 9 at day 25 of differentiation (differentiation replicate 22) - 3 wild type kidney organoids, 3 organoid generated from a male patient with *COL4A4/COL4A5* variants, and 3 organoids generated from a female patient with *COL4A4/COL4A5* variants (in **Methods**, ***SDS-PAGE and immunoblotting***, pg. 16).

**Replicates**

- You should report how often each experiment was performed
- You should include a definition of biological versus technical replication
- The data obtained should be provided and sufficient information should be provided to indicate the number of independent biological and/or technical replicates
- If you encountered any outliers, you should describe how these were handled
- Criteria for exclusion/inclusion of data should be clearly stated
- High-throughput sequence data should be uploaded before submission, with a private link for reviewers provided (these are available from both GEO and ArrayExpress)

Please outline where this information can be found within the submission (e.g., sections or figure legends), or explain why this information doesn’t apply to your submission:

**Mass spectrometry:** For each proteomic study (mouse and organoids), we had all samples analyzed in one run (one technical replicate). For the organoids, we had 3 independent biological replicates (i.e., 3 different samples) per time-point (in **Methods**, ***section laser microdissection microscopy*** pg. 17), and for the mouse studies we had 6 independent biological replicates for the fractionation (in **Methods**, ***section laser microdissection microscopy*** pg. 17), and 4 biological replicates for the laser microdissection microscopy experiments (in this, for each sample we collected 150 glomerular sections; in **Methods**, ***section laser microdissection microscopy*** pg. 17).

**Immunofluorescence**: immunofluorescence microscopy and histological staining experiments were performed at least twice (technical replicate); negative controls (secondary antibody only) were used for all immunofluorescence assays (in **Methods**, ***Histology and immunofluorescence***, pg. 15).

**SDS-PAGE and immunoblotting**: western blot was performed once, using 3 independent biological replicates (3 samples, for which 3 independent technical replicates were used; in **Methods**, ***SDS-PAGE and immunoblotting***, pg. 16).

**Statistical reporting**

- Statistical analysis methods should be described and justified
- Raw data should be presented in figures whenever informative to do so (typically when N per group is less than 10)
- For each experiment, you should identify the statistical tests used, exact values of N, definitions of center, methods of multiple test correction, and dispersion and precision measures (e.g., mean, median, SD, SEM, confidence intervals; and, for the major substantive results, a measure of effect size (e.g., Pearson's r, Cohen's d)
- Report exact p-values wherever possible alongside the summary statistics and 95% confidence intervals. These should be reported for all key questions and not only when the p-value is less than 0.05.

Please outline where this information can be found within the submission (e.g., sections or figure legends), or explain why this information doesn’t apply to your submission:

Statistical analysis was carried out within Proteome Discoverer using an in-built Two-way ANOVA test with post-hoc Benjamini-Hochberg correction. Principal component analysis (PCA) and unsupervised hierarchical clustering based on a Euclidean distance-based complete-linkage matrix were performed using Rstudio (v. 1.2.5042, http://rstudio.com) with the ggplot2 package (v.3.3.2, https://ggplot2.tidyverse.org) that was also used to generate PCA plots and heat maps. For the integrated proteomic analysis, previously published young human glomerular and kidney tubulointerstitial data (PRIDE accession PXD022219) was re-processed with Proteome Discoverer to allow direct comparisons with newly acquired data. Then, kidney organoid, mouse and human proteomics datasets were compared using Spearman Rank correlation. Dataset comparisons, for both cellular and ECM cellular fractions, were performed separately for the matrisome proteins only and basement membrane proteins only. The ComplexHeatmap package (v2.2.0, (Gu et al., 2016); http://bioconductor.org/packages/release/bioc/html/ComplexHeatmap.html) was used to generate correlation plots (in **Methods**, ***Statistical*** ***analysis***, pg. 20)

(For large datasets, or papers with a very large number of statistical tests, you may upload a single table file with tests, Ns, etc., with reference to sections in the manuscript.)

**Group allocation**

- Indicate how samples were allocated into experimental groups (in the case of clinical studies, please specify allocation to treatment method); if randomization was used, please also state if restricted randomization was applied
- Indicate if masking was used during group allocation, data collection and/or data analysis

Please outline where this information can be found within the submission (e.g., sections or figure legends), or explain why this information doesn’t apply to your submission:

N/A

**Additional data files (“source data”)**

- We encourage you to upload relevant additional data files, such as numerical data that are represented as a graph in a figure, or as a summary table
- Where provided, these should be in the most useful format, and they can be uploaded as “Source data” files linked to a main figure or table
- Include model definition files including the full list of parameters used
- Include code used for data analysis (e.g., R, MatLab)
- Avoid stating that data files are “available upon request”

Please indicate the figures or tables for which source data files have been provided:

This project contains the following underlying data hosted at Figshare: https://doi.org/10.6084/m9.figshare.c.5429628

Figure 1 Original IF Images: B Whole-mount immunofluorescence for kidney cell types; F Representative whole mount immunofluorescence images of wild-type and Alport kidney organoids; G Immunofluorescence for LAMB2.

Figure 1 Original light microscope Images: C Representative photomicrographs of day 18 kidney organoids (left) and human and mouse fetal kidneys (right).

Figure 1 Original TEM Images: D Transmission electron micrographs of tubular BM in day 25 kidney organoid and E19 mouse fetal kidney.

Figure 1 Original western blotting image: H Immunoblotting for LAMB2 using total lysates from wild type and Alport organoids.

Figure 2 Original IF Images: A Confocal immunofluorescence microscopy of wild-type kidney organoids; B perlecan and nidogen on days 11, 18 and 25 of differentiation.

Figure 4 Original IF Images: A Immunofluorescence for key type IV collagen and laminin isoforms showing their emergence and distribution in kidney organoid BM; D Immunofluorescence for specific collagen IV isoforms in maturing glomeruli in E19 mouse kidney and in glomerular structures (indicated by dashed lines) in day 25 organoids.

This project contains the following extended data:

Figure S1 Morphological characteristics of wild-type kidney organoids, fetal human kidney, and Alport kidney organoids.

Figure S2 Time course proteomic analysis of kidney organoid differentiation.

Figure S3 Single cell-RNA sequencing data analysis of human kidney organoids.

Figure S4 Proteomic analysis of E19 mouse fetal kidney and correlational comparison with kidney organoid proteomics.

Figure S5 Single-cell RNA sequencing analysis of human fetal kidney.

Figure S6 Integrated correlational analysis of organoid and in vivo kidney datasets.

Table S1 Human fetal kidney and hiPSC general information
